
JOURNAL INFORMATION
==============================
NLM Title Abbreviation: ScientificWorldJournal
Journal ID: ScientificWorldJournal
NLM Journal ID: 101131163
Journal ID: TSWJ

The Scientific World Journal

ISSN: 2356-6140
EISSN: 1537-744X
Publisher: Wiley

ARTICLE INFORMATION
==============================
PMCID: PMC6083986
PMID: 30147594
DOI: 10.1100/tsw.2001.130
Article ID: 180268
Article version: 1
Subjects: Short Report

Synergistic Effects of DNA and Cytoskeleton Damage-Inducing Agents on Apoptosis in Human Tumor Cell Lines

Shang Xueyuan *
Fujita Yasuko
Oka Syuichi
Yamazaki Yoshimitsu
Biosignaling Department, National institute of Bioscience and Human Technology, Agency of Industrial Science and Technology, 1-1 Higashi, Tsukuba, Ibaraki 305-8566, Japan
*Xueyuan Shang: xshang@nibh.go.jp
Publication date: 2001
Electronic publication date: 2001 Feb 2
Volume: 1
First page: 110
Last page: 110
Copyright: Copyright © 2001 Xueyuan Shang et al.
Copyright year: 2001
License: This is an open access article distributed under the Creative Commons Attribution License, which permits unrestricted use, distribution, and reproduction in any medium, provided the original work is properly cited.
License URL: https://creativecommons.org/licenses/by/3.0/

==============================
Miami *Nature Biotechnology* Short Reports
*TheScientificWorld* (2001) 1(S3), 110SR
ISSN 1532-2246; DOI 10.1100/TSW.2001.130

### SYNERGISTIC EFFECTS OF DNA AND CYTOSKELETON DAMAGE-INDUCING AGENTS ON APOPTOSIS IN HUMAN TUMOR CELL LINES

Xueyuan Shang*, Yasuko Fujita, Syuichi Oka, and Yoshimitsu Yamazaki

Biosignaling Department, National institute of Bioscience and Human Technology, Agency of Industrial Science and Technology, 1-1 Higashi, Tsukuba, Ibaraki 305-8566, Japan
* xshang@nibh.go.jp

**INTRODUCTION.** Actinomycin D (AD) is a common DNA-damaging agent. We have recently reported that AD-induced apoptosis in human megakaryoblastic leukemia cell line CMK-7 was greatly accelerated by concomitantly added colcemid (CL), a cytoskeleton-damaging agent (1). The present work was conducted to investigate the synergistic enhancement of apoptosis by the combination of three different DNA-damaging agents AD, etoposide (VP-16) and mitomycin C (MMC) with cytoskeleton-damaging agents colcemid (CL), cytochalasin D (CD) and vinblastin (Vin) in different human tumor cell lines.

**METHOD.** Human leukemia cell lines CMK-7, U937, Molt-4 and K562 and solid tumor cell lines Hela S3 and Colo 320DM were used for the experiment. Cells were treated for a definite time and then were collected for caspase-3, Tunel and viability assay. Caspase-3 activity in the cell lysate was measured using a caspase-3 kit. Fluorescence at 440nm was measured by excitation at 380nm with a Hitachi RF-5000 spectrofluorophotometer. Tunel assay for leukemia cell lines: DNA cleaved ends were labeled with the APO-direct kit, the stained cells were analyzed with a flow cytometer. For solid tumor cell lines, an in situ apoptosis detection kit was used. Trypan blue method was used to measure cell viability.

**RESULTS. 1**. Combination of AD with different cytoskeleton-damaging agents: When AD was combined with CL or CD or Vin, a great enhancement in activation of caspase-3 was observed in leukemia cell lines as well as solid tumor cell lines. Results of AD plus CL or CD or Vin for Tunel assay and cell viability were parallel to the caspase-3 data. **2**. Combination of VP-16 with different cytoskeleton-damaging agents: in leukemia cell lines U937, MOLT-4, K562 (CMK-7 not tested), enhancements in caspase-3 activation were obvious. In the solid tumor Hela S3 cell line, there was a slight enhancement in caspase-3 activity. But there was no significant synergistic enhancement in caspase-3 activity in the Colo 320DM cell line. **3**. Combination of MMC with different cytoskeleton-damaging agents: There were obvious enhancements in caspase-3 activation in K562, U937 and Hela S3 cell lines and only slight or insignificant synergistic enhancement in caspase-3 activity in MOLT-4 and Colo 320DM cell lines.

**DISCUSSION.** Apoptosis plays an important role in cancer chemotherapy. The synergistic effect of chemotherapeutic agents is very exciting and interesting. The synergistic effect makes it possible to reduce dose of drugs and their toxicity and raise the efficiency of the treatment. In DNA-damage-induced apoptosis, combination with cytoskeleton-damaging agents showed synergistic effect. DNA-damaging agent-induced apoptosis is very important for the synergistic effects. AD is a potent apoptosis inducer. Its apoptosis inducing effect was greatly enhanced by different cytoskeleton damaging agents. Compared with AD, the apoptosis induced by MMC is weak and slow. The synergistic effect between MMC and cytoskeleton-damaging agents is weak. In some cell line, there was no synergistic effect between MMC and cytoskeleton damaging agents.

**REFERENCE.**

1. Yamazaki, Y., Tsuriga, M., Zhou, D., Fujita, Y., Shang, X., Dang, Y., Kawasaki, K., and Oka, S. (2000) Exp. Cell Res. 259, 64-78
